# Assessing self-reported core competencies of public health practitioners in Lebanon using the WHO-ASPHER validated scale: a pilot study

**DOI:** 10.1186/s12909-022-03940-4

**Published:** 2022-12-20

**Authors:** Katia Iskandar, Chadia Haddad, Aline Hajj, Hala Sacre, Rony M. Zeenny, Marwan Akel, Pascale Salameh

**Affiliations:** 1 INSPECT-LB (Institut National de Santé Publique, d’Épidémiologie Clinique Et de Toxicologie-Liban), Beirut, Lebanon; 2 Faculty of Pharmacy, Lebanese University, Beirut, Lebanon; 3 School of Health Sciences, Modern University for Business and Science, Beirut, Lebanon; 4 Beirut, Lebanon; 5 Research Department, Psychiatric Hospital of the Cross, Jal El Dib, Lebanon; 6 School of Medicine, Lebanese American University, Byblos, Lebanon; 7 Laboratoire de Pharmacologie, Pharmacie Clinique Et Contrôle de Qualité Des Médicament, Faculty of Pharmacy, Saint-Joseph University of Beirut, Beirut, Lebanon; 8 Faculty of Pharmacy, Université Laval, Québec, Canada; 9 Oncology Division, CHU de Québec- Université Laval Research Center, Québec, Canada; 10 Department of Pharmacy, American University of Beirut Medical Center, Beirut, Lebanon; 11 School of Pharmacy, Lebanese International University, Beirut, Lebanon; 12 Department of Education, Lebanese International University, Beirut, Lebanon; 13 Department of Primary Care and Population Health, University of Nicosia Medical School, 2417 Nicosia, Cyprus

**Keywords:** Competencies, Public Health, Scale, Validation, WHO-ASPHER

## Abstract

**Background:**

The World Health Organization and the Association of Schools of Public Health in the European Region recommend the self-assessment of public health core competencies to strengthen the proficiency of the public health workforce and prepare them for future challenges. A framework for these competencies is lacking and highly needed in Lebanon. This study aims to validate the WHO-ASPHER self-declared scale and evaluate the perceived competency level of the different categories of Lebanese public health practitioners.

**Methods:**

This population-based cross-sectional study conducted online between July and September 2021 involved 66 public health practitioners who graduated from different universities in Lebanon. Data were collected using the snowball technique via a self-report questionnaire that assessed public health proficiency, categorized into 1) content and context, 2) relationship and interactions, and 3) performance and achievements. The rotated component matrix technique was used to test the construct validity of the scales. Bivariate and multivariate analyses were performed after ensuring the adequacy of the models. Significance was set at a *p*-value < 0.05.

**Results:**

The factor analysis for scale domains showed that the Barlett test sphericity was significant (*p* < 0.001), high loadings of items on factors, and Cronbach’s alpha values of more than 0.9 in all three categories, showing an appropriate scale validity and reliability. The perceived level of competencies was significantly different between public health professionals and other health professionals with public health activities. All respondents scored low in most public health categories, mainly science and practice.

**Conclusion:**

Data findings showed variability of self-declared gaps in knowledge and proficiency, suggesting the need to review the national public health education programs. Our study offers a valuable tool for academia and public health professionals to self-assess the level of public health proficiency and guide continuous education needs for professional development.

**Supplementary Information:**

The online version contains supplementary material available at 10.1186/s12909-022-03940-4.

## Background

Public health is an organized societal effort based on different structures and processes intended to understand, safeguard and improve population health and reduce health inequalities [1–3]. It is the art of applying science in the context of politics to assess the influences of health systems and interventions on societies’ mental and physical health promotion and efficiency, health protection, and disease prevention [1–8]. Public health tackles all socioeconomic, political, physical, chemical, and biological conditions that impact or interact with the population’s health [9]. A high-performing public health system requires a competent public health workforce with adequate baseline capacity and transferrable skills to be held professionally accountable for the health of a defined population [9–14]. Therefore, a lack of workforce competence contributes to substandard service delivery [15] and leads to social, economic, and health burdens [14–16]. Alternatively, strengthening the performance and core competencies contributes to the sustainable development of nations [14, 17].

To ensure a high level of proficiency and highlight the gaps in knowledge that need strengthening, self-assessment of core competencies in public health is considered a starting point. The baseline requirements for high-level public health performance and service delivery differ between countries [18]. More than ten frameworks for assessing core competencies in public health are available for use, originating from different countries such as the United States of America (USA), Canada, New Zealand, the United Kingdom, and other European countries [9, 19–28]. The knowledge and skills needed to carry out core professional functions in public health are complex [9, 10, 20].

Published studies used mainly a formulated survey to assess the perceived needs of public health practitioners for training and identify gaps in knowledge [29–31]. A recent review of the questions asked in 24 published articles showed a lack of consistency, thus limiting the generalizability of the findings [32]. Another systematic review published in 2012 evaluated 126 public health workforce articles and gray literature and recommended the development of quantifiable output measures to offer baseline data to build models that address workforce demand [33]. This finding highlights the need for a country-specific framework for the self-assessment of public health core competencies to overcome these barriers.

Consequently, in the absence of requirements for health workers to receive public health training and the lack of preset national core competencies to assess the competence of the public health workforce, matching population health priorities and professional competencies is very challenging [26]. The World Health Organization (WHO) and the Association of Schools of Public Health in the European Region (ASPHER) set a context-specific core competency framework designed to assess the gaps and weaknesses in the levels of knowledge, skills, aptitudes of public health practitioners, aiming to strengthen public health workforce [26]. The framework provides level descriptors to interpret the extent to which competencies are mastered based on the Dreyfus model of adult skill acquisition [34]. The WHO framework sets three categories of competency needed to assess the extent of mastered competencies in each domain [26]. Category 1 evaluates the science and practice, health promotion, one-health, and security; it also tackles law, policies, and ethic-related frameworks that reinforce public health practice. Category 2 examines the level of competencies in terms of relations and interactions, such as communication and advocacy, collaboration and partnership, and leadership and system thinking. Category 3 addresses performance and achievements, such as professional development, governance, ethical practice, and resource management [26].

The assessment of competencies offers a broader perspective on how to serve the needs of populations and create people-centered services. It also helps improve the curricula and continuing professional development based on existing capacity and training requirements [26, 35].

Furthermore, lessons learned from the COVID-19 pandemic highlighted the gaps in global health systems readiness facing this threat and the need to strengthen the core competencies of the health workforce to deliver efficient public health functions [35–38]. More specifically, in Lebanon, the pandemic and the Port of Beirut explosion on August 4, 2020, revealed a chaotic Lebanese health system, struggling to manage these concomitant public health crises with limited or lack of resources, drug shortages, a damaged infrastructure, health professionals’ migration, and economic downturn [39]. This challenging situation shows the need for a national health system plan for humanitarian crises, relying on a highly competent and trained public health workforce. The public health workforce (PHW) is highly diverse and complex [40], including a broad range of occupational backgrounds trained in a variety of institutional settings involved in the protection and promotion of public health [40].

To our knowledge, little is known about the competencies of public health professionals in Lebanon. Public health education is delivered in schools/faculties of health sciences and/or health professions. Degrees offered can be undergraduate or graduate and can be professionally oriented or research-driven (i.e., to be completed by a PhD). Public health professionals work in public and private sectors (non-governmental organizations and health institutions), while some teach in universities. The only professional association for public health workers in Lebanon is the Lebanese Epidemiological Association (LEA), which has been providing an umbrella to academic and field workers in epidemiology and public health in Lebanon since 1994. However, it does not have guidelines related to the job market of public health professionals and does not give directions regarding national educational needs in the field.

This study primarily aims to validate the public health self-assessment competency scale adapted from the WHO-ASPHER framework and assess the self-declared competencies of Lebanese public health professionals using a validated scale. The results would help determine the gaps in knowledge, prioritize the domains that need strengthening in public health, and identify the national public health educational program needs and necessary competencies for prospective public health bachelor or master graduates.

## Methods

### Study design and sampling

A population-based cross-sectional study conducted online between July 01, 2021, and September 30, 2021, involved 66 public health practitioners who graduated from different universities in Lebanon. Data were collected using the snowball technique via a self-report questionnaire developed on Google Forms (https://forms.gle/J4wXjq5sZUBYdqfR7) and shared on social media (WhatsApp, Facebook, and LinkedIn) of healthcare professional groups and public health graduates from different universities (Additional file 1 Appendix 1). Public health graduates and practitioners, healthcare professionals involved in public health activities in Lebanon, and epidemiologists were eligible to participate in the study.

### Ethics approval

The Lebanese International University research committee approved this study (2020RC-047-LIUSOP). The objectives were stated on the landing page of the survey, and participants had to consent to participate before enrolling. They received no compensation in return for their participation, which was entirely voluntary.

### Sample size calculation

The G-power 3.1.9.4 software [41] calculated a minimum sample of 64 participants based on a Cohen effect size *f*^*2*^ = 30% (large explanation of the dependent variable by the model variables), an alpha error of 5%, a power of 80%, and considering ten factors to be entered in the multivariable analysis.

### Questionnaire (Appendix 1)

The online survey tool was in English and included closed-ended questions. It was inspired by published articles and reports [14, 19, 25] and adapted by the authors (of whom three are public health experts) to fit the Lebanese context of public health practice. Some items were clarified by adding the geographical location “in Lebanon”, while others were removed or adapted to the Lebanese practice.

The questionnaire consisted of four main sections. The first section covered sociodemographic characteristics (age, gender, area of residence, specialization field, public health practice domain, and years of experience). The second section consisted of public health essential operations, and the third section assessed the level of public health workforce competency (detailed below). In the fourth section, public health practitioners gave feedback on their experience by rating 15 statements on a 5-point Likert scale ranging from totally disagree to totally agree. The five options were collapsed into three categories as follows: strongly agree/agree, neutral, disagree/strongly disagree.

#### Competency assessment section

Competency assessment items were distributed over three main categories, each composed of several domains, as presented by the WHO-ASPHER framework [26]:

1. Content and context. This category encompasses four domains: 1) Science and practice; 2) Promoting health; 3) Law, policies, and health services; 4) One-health and health security.
2. Relations and interactions. This category encompasses three domains: 1) Leadership and systems thinking; 2) Collaboration and partnerships; 3) Communication, culture, and advocacy.
3. Performance and achievements. This category encompasses three domains: 1) Governance and resource management; 2) Professional development and reflective ethical practice; 3) Organizational literacy and adaptability.

Participants were asked to rate their perceived level of proficiency on each competency statement in the three categories listed above [26] on a 4-point Likert scale: 1 (none: I am unaware or have very little knowledge of the skill), 2 (aware: I have heard of, but have limited knowledge or ability to apply the skill), 3 (knowledgeable: I am comfortable with my knowledge or ability to apply the skill), and 4 (proficient: I am very comfortable, am an expert, or could teach this skill to others). The average score for each category represents the total number of allocated scores per statement divided by the total number of statements per category. The results represent the average score for all domains. A score of 1–2 per domain means a low level of competency that needs strengthening, while a score of 3–4 is interpreted as a high level of competency [26].

### Statistical analysis

Data were extracted from Google on an Excel spreadsheet and analyzed using SPSS version 25.0. A descriptive analysis evaluated the sample demographic characteristics using the absolute frequencies and percentages for categorical variables and means and standard deviations (SD) for quantitative measures.

The rotated component matrix technique was used to test the construct validity of the scales. The Kaiser–Meyer–Olkin’s (KMO) measure of sampling adequacy and Bartlett’s test of sphericity were calculated to ensure the adequacy of the model [42]. Factors with eigenvalues values of more than one were retained, and the scree plot method was used to determine the number of components to extract [43]. Only items with factor loading greater than 0.4 were considered [44]. Cronbach’s alpha was calculated to determine the internal consistency of the scale.

For bivariate analysis, the Chi-square test and the Fisher exact test were used to compare percentages, and the Student T-test and the Mann Whitney were applied to compare means between two groups. The multivariate analysis of covariance (MANCOVA) was performed, considering the competency item per category as the dependent variable and the public health specialty versus others as the independent variable after adjusting for gender, years of experience, area of residence, and area of practice. Adjusted coefficients (beta) and their 95% confidence intervals served to interpret the associations between the dependent and independent variables. Residual plots were used to assess the assumptions of the MANCOVA (homoscedasticity); the linear relationship between the continuous dependent and the independent variables was ensured, in addition to the absence of interaction and co-linearity. In all cases, a value of *p* < 0.05 was considered significant.

## Results

### Characteristics of the participants

Table 1 summarizes the sociodemographic characteristics of the study sample. Participants had a mean age of 29.74 ± 7.57 years, were predominantly females (84.8%), mainly living in Mount Lebanon (59.1%), with five or fewer years of experience (71.2%). Study degrees were distributed as follows: Bachelor of Science (BS) in public health (33.3%), pharmacy (21.2%), nursing (10.6%), nutrition (10.6%), and medicine (3%). The vast majority of the respondents practiced in more than one area (63.6%). The fields of practice included academia (63.6%), research epidemiology (57.6%), non-governmental organizations (NGOs) (47%), Ministry of Public Health (37.9%), and medical settings (36.4%), added to fresh graduates with a degree in public health (21.2%).

**Table 1:** Sociodemographic and other characteristics of the participants (*n* = 66)

Variable	n (%)
**Gender**	
Male	10 (15.2%)
Female	56 (84.8%)
**Area of residence**	
Beirut	18 (27.3%)
Mount Lebanon	39 (59.1%)
Other region (North, south, Bekaa)	9 (13.6%)
**Years of experience**	
1 – 5 years	47 (71.2%)
6 – 10 years	10 (15.2%)
More than 10 years	9 (13.6%)
**Basic specialty degree**	
BS in Public health	22 (33.3%)
Pharmacy	14 (21.2%)
Nursing	7 (10.6%)
Nutrition	7 (10.6%)
Other	16 (23.2%)
**Area of practice**^**a**^	
Academia	42 (63.6%)
Medical setting	24 (36.4%)
Research epidemiology	38 (57.6%)
NGO	31 (47.0%)
MOPH	25 (37.9%)
Fresh graduate	14 (21.2%)
	**Mean ± SD**
**Age** (years)	29.74 ± 7.57

*Abbreviations*: *BS* bachelor of sciences, *MOPH* Ministry of Public Health, *n* number of participants, *NGO* non-governmental organization, *SD* standard deviation

^a^The same person could have several areas of practice

### Factor analysis of the WHO-ASPHER competency scale

A factor analysis was performed to assess the validity of the public health competency scale and the adequacy of the model.

For the “Content and Context” category, the KMO measure of sampling adequacy was 0.923 for “Science and Practice”, 0.924 for “Promoting Health”, 0.915 for “Law, Policies, and Health Services”, and 0.972 for “One-Health and Health Security”. Regarding “Science and Practice”, the first factor explained the most variance by 69.97%, followed by 8.71% for the second factor. For “Promoting Health”, “Law, Policies, and Health Services”, and “One Health and Health Security”, the first factor explained all the variances by 76.16%, 81.91%, and 77%, respectively (Table 2A).

**Table 2:** Factor analysis of public health competencies according to categories and domains

A: Promax rotated matrix, for category 1: Content and Context				
**Science and Practice domain**				
** Factor**	**Item**	**Factor 1**		**Factor 2**
Identify the strengths and weaknesses of routine data and use these data as part of the complex assessment of population needs	4	1.073		
Determine the key features of the epidemiology, trends, incidence, and prevalence of the significant diseases in Lebanon	2	0.833		
Address the main health needs of the Lebanese population	6	0.832		
Retrieve, analyze, and appraise evidence from all data sources to support decision-making	5	0.817		
Describe the features of national demographic structure and its implications for public health	1	0.799		
Use vital statistics and health indicators	3	0.798		
Compare and assess the needs and services provided to meet health needs	8	0.785		
Establish and monitor indicators of population health	7	0.766		
Contribute to or lead community-based health needs assessments	9	0.598		
Show a high level of knowledge of research methods and analysis techniques	12			1.055
Design and conduct qualitative and/or quantitative research that adds to the evidence base for public health practice	11			0.951
Review routine data and the literature to what actions should be taken to meet health needs	10			0.787
Evaluate local public health services and interventions, applying sound methods based on recognized evaluation models	13			0.692
**Percentage variance explained**	78.68	69.97		8.71
**Cronbach alpha = 0.964**				
**Kaiser–Meyer–Olkin (KMO) = 0.923**				
**Bartlett’s test of sphericity *p* < 0.001**				
**Promoting Health domain**				
**Factor**		**Item**		**Factor 1**
Know the rationale for screening programs and the basis of secondary prevention in my country		9		0.919
Use health promotion theory and the options for delivering health-promotion initiatives		1		0.897
Challenge incorrect information delivered to the public using a wide range of approaches, including communication with the media and politicians		8		0.897
Promote the health of the public using evidence-based methods		3		0.886
Raise health literacy		2		0.876
Ensure that health education and health literacy activities are informed by evidence and/or theory		4		0.875
Contribute to the evaluation of the effectiveness of activities to promote health to lead changes at various levels across different sectors		5		0.872
Use appropriate methods to foster citizens empowerment and community engagement		6		0.864
Consult with the public to engage meaningful decision-making that represents the wider societal views		7		0.855
Focus on disease prevention, reduction of inequalities, and equity in access to health services		10		0.849
Explore the underlying causes of morbidity and mortality, and recommendations to address these determinants of health and health services		11		0.805
**Percentage variance explained**		76.16%		
**Cronbach alpha = 0.968**				
**Kaiser–Meyer–Olkin (KMO) = 0.924**				
**Bartlett’s test of sphericity *p* < 0.001**				
**Law, Policies, and Health Services domain**				
**Factor**		**Item**		**Factor 1**
Develop and implement strategies based on relevant evidence, legislation, emergency planning, procedures regulations, and policies		6		0.927
Contribute to the delivery of equitable and effective health care and policies to improve the health of the public		5		0.923
Maximize opportunities to protect and promote health and well-being using applied laws and regulations		7		0.914
Comply with the legislation and professional codes of practice in my interaction with others		1		0.910
Understand and apply the laws and regulations directly or indirectly applicable to the practice of public health in Lebanon		2		0.903
Apply scientific principles and concepts to inform discussion of health-related fiscal, social, and political issues		3		0.886
Compare and contrast health and social service delivery systems between countries		4		0.871
**Percentage variance explained**		81.91%		
**Cronbach alpha = 0.962**				
**Kaiser–Meyer–Olkin (KMO) = 0.915**				
**Bartlett’s test of sphericity *p* < 0.001**				
**One Health and Health Security domain**				
**Factor**		**Item**		**Factor 1**
Comply with the requirements of both formal and informal surveillance systems and conduct risk assessment		9		0.911
Prevent risks and mitigate the health crises that originate at the interface between human, animals, and environments and affect the health of the population		2		0.902
Apply the International Health regulations to coordinate and develop strategic partnerships and resources in key sectors and disciplines for health security purposes		5		0.892
Understand the impact of climate on health and the responsibility of public health for protecting the natural environment		12		0.891
Analyze critically the changing nature, key factors, and resources that shape One Health		3		0.891
Promote occupational health and health and safety regulations and legislations		6		0.887
Identify and describe environmental determinants of health and connections between environmental protection and public health policy		11		0.882
Use multisectoral evidence-based guidelines for preventing and controlling health risks and diseases		8		0.881
Understand the One Health		4		0.875
Identify and assure minimum safety standards in delivering services		10		0.860
Understand the local implications of the One Health approach and its global interconnectivity		1		0.859
Apply the practical principles of food safety essential to public health		7		0.793
**Percentage variance explained**		77.00%		
**Cronbach alpha = 0.972**				
**Kaiser–Meyer–Olkin (KMO) = 0.911**				
**Bartlett’s test of sphericity *p* < 0.001**				
**B: Category 2: Relations and Interactions**				
**Factor analysis, promax rotated matrix for Category 2: Leadership and Systems Thinking domain**				
**Factor**		**Item**	**Factor 1**	
Catalyze behavioral, and/or cultural changes		7	0.938	
Lead and work as part of an interdisciplinary team		6	0.936	
Support initiatives for change at the organization, community, or individual level		8	0.935	
Understand principles of systems thinking to the improve delivery of public health services		9	0.926	
Facilitate the development of other leaders		2	0.922	
Identify and support the roles and responsibilities of all team members, including external stakeholders		3	0.922	
Show practicality, flexibility, and adaptability in working with others to achieve public health goals		5	0.918	
Demonstrate emotional intelligence and understand the impact of one’s belief, values, and behaviors on decision-making and others’ reactions		4	0.914	
Motivate others to work toward common vision, program, and/or organizational goals		1	0.886	
**Percentage variance explained**		85.04%		
**Cronbach alpha = 0.978**				
**Kaiser–Meyer–Olkin (KMO) = 0.920**				
**Bartlett’s test of sphericity *p* < 0.001**				
**Collaboration and Partnerships domain**				
**Factor**		**Item**	**Factor 1**	
Evaluate partnerships and address barriers to successful collaboration to improve public		5	0.943	
Build, maintain, and effectively use strategic alliances, coalitions, professional networks, and partnerships to plan and generate evidence implement programs		4	0.935	
Establish effective partnerships and understand the priorities and motivations of a wide range of stakeholders		2	0.934	
Identify, connect, and manage relationships with stakeholders in interdisciplinary and intersectoral projects to improve public health services and goals		3	0.917	
Understand and apply effective techniques for working with boards and governance		6	0.916	
Work across sectors in organizational structures at the national and international levels		1	0.846	
**Percentage variance explained**		83.88%		
**Cronbach alpha = 0.961**				
**Kaiser–Meyer–Olkin (KMO) = 0.880**				
**Bartlett’s test of sphericity *p* < 0.001**				
**Communication, Culture, and Advocacy domain**				
**Factor**		**Item**	**Factor 1**	
Understand and apply cultural awareness and sensitivity in communication with diverse populations		5	0.938	
Communicate with respect when representing professional opinions, and encourage other team members		6	0.935	
Recognize that social media and social marketing are increasingly important tools		4	0.927	
Deliver administrative tasks that require communication within or across organizations		8	0.919	
Advocate for health-related public policies and services to promote and protect human health and well-being		9	0.901	
Prepare a meeting agenda		7	0.900	
Convey information and complex scientific evidence in an understandable way to people		3	0.896	
Communicate strategically by defining target audience, listening, and developing audience-appropriate messaging		1	0.894	
Understand the importance of communication at different organizational levels to gain political commitment, policy support, and social acceptance for a health goal or program		2	0.886	
**Percentage variance explained**		82.94%		
**Cronbach alpha = 0.974**				
**Kaiser–Meyer–Olkin (KMO) = 0.917**				
**Bartlett’s test of sphericity *p* < 0.001**				
**C—Category 3: Performance and achievements**				
**Factor analysis, promax rotated matrix for Category 3: Governance and Resource Management domain**				
**Factor**		**Item**	**Factor 1**	
Design proactively and monitor quality standards and apply quality improvement methods and tools to ensure that quality standards are met		7	0.916	
Demonstrate knowledge of basic business practices and develop a business plan		6	0.899	
Use risk management principles and programs		9	0.888	
Develop descriptions to assure staffing at various organization levels		4	0.869	
Use key accounting principles and financial management tools		8	0.869	
Plan the allocation of work tasks to achieve the goals set by the organization		3	0.853	
Understand and apply the principles of economic thinking in public health		10	0.843	
Perform health evaluation and assessment of a given procedure, intervention strategy, or policy		11	0.840	
Conduct hiring interviews and evaluate candidates		5	0.832	
Apply knowledge of organizational systems, theories, and behaviors to set priorities for resources and achieve clear strategic goals and objectives		1	0.803	
Manage people effectively by providing clarity on task responsibility, provide training, and give regular feedback on performance		2	0.793	
**Percentage variance explained**		73.23%		
**Cronbach alpha = 0.963**				
**Kaiser–Meyer–Olkin (KMO) = 0.915**				
**Bartlett’s test of sphericity *p* < 0.001**				
**Professional Development & Reflective Ethical Practice domain**				
**Factor**		**Item**	**Factor 1**	
Ensure the availability of development opportunities		5	0.950	
Act and promote evidence-based professional practice		7	0.949	
Demonstrate an ability to understand and manage conflict-of-interest situations		6	0.947	
Act according to ethical standards and norms with integrity, and promote professional accountability, social responsibility, and the public health good		3	0.943	
Demonstrate willingness to pursue learning in public health		1	0.932	
Address your own development needs based on career goals and required competencies		2	0.931	
Critically review and evaluate your own practices in relation with public health principles		4	0.900	
**Percentage variance explained**		87.65%		
**Cronbach alpha = 0.976**				
**Kaiser–Meyer–Olkin (KMO) = 0.856**				
**Bartlett’s test of sphericity *p* < 0.001**				
**Organizational Literacy and Adaptability domain**				
**Factor**		**Item**	**Factor 1**	
Demonstrate persistence, perseverance, resilience, and the ability to call on personal resources and energy at time of challenge		2	0.933	
Show entrepreneurial orientation through proactiveness, innovativeness, and risk-taking, generating potential solutions to critical situations		3	0.914	
Apply for available funding sources and opportunities		5	0.907	
Cope with uncertainty and manage work-related stress		1	0.905	
Respond to call for project applications and grants		6	0.904	
Adapt to changing professional environments and circumstances		4	0.894	
Draft tender and project briefs		7	0.882	
**Percentage variance explained**		82.02%		
**Cronbach alpha = 0.963**				
**Kaiser–Meyer–Olkin (KMO) = 0.918**				
**Bartlett’s test of sphericity *p* < 0.001**				

Regarding the “Relations and Interactions” category, the KMO measure of sampling adequacy was 0.920 for the “Leadership and Systems Thinking”, 0.880 for “Collaboration and Partnerships”, and 0.917 for “Communication, Culture, and Advocacy”. For the “Leadership and Systems Thinking”, “Collaboration and Partnerships”, and “Communication, Culture, and Advocacy”, the first factor explained all the variances by 85.04%, 83.88%, and 82.94%, respectively (Table 2B).

Finally, in the “Performance and Achievements” category, the KMO measure of sampling adequacy was 0.915 for “Governance and Resource Management”, 0.856 for “Professional Development and Reflective Ethical Practice”, and 0.918 for “Organizational Literacy and Adaptability”. For the “Governance and Resource Management”, “Professional Development and Reflective Ethical Practice”, and “Organizational Literacy and Adaptability”, the first factor explained all the variances by 73.23%, 87.65%, and 87.02%, respectively. In all categories, Barlett’s test of sphericity was significant (*p* < 0.001), and Cronbach’s alpha value was higher than 0.9 (Table 2C).

### Essential operations in public health

Table 3 describes the perceived level of knowledge for public health essential operations. Most participants declared being knowledgeable of the public health essential operations. Almost half of them (48.5%) considered they had adequate knowledge in assuring sustainable organizational structures and financing.

**Table 3:** The level of knowledge for the statement of public health essential operations

	**Frequency (%)**
Surveillance of population health and well-being	42 (63.6%)
Monitoring and response to health hazards and emergencies	41 (62.1%)
Health protection, including environmental, occupational, food safety, and other	46 (69.7%)
Health promotion, including action to address social determinants and health inequity	48 (72.7%)
Disease prevention, including early detection of illness	44 (66.7%)
Assuring governance for health and well-being	39 (59.1%)
Assuring a sufficient and competent health workforce	39 (59.1%)
Assuring sustainable organizational structures and financing	32 (48.5%)
Advocacy communication and social mobilization for health	40 (60.6%)
Advancing public health research to inform policy and practice	44 (66.7%)

### Bivariate analysis

#### Competency levels between specialties

Table 4 shows the differences in competency levels between all specialties and between public health professionals versus all the others. Overall, graduates with a BS in public health reported a lower competency compared to other specialties in most categories and domains, with percentages varying by 2 to 4 folds.

**Table 4:** Differences in the levels of competencies between public health and other specialties

	**Public health with BS vs other specialties**		**All the specialties**						
	**Public health with BS degree**	**Other Specialties**	**Pharmacist**	**Nursing**	**Nutrition**	**Medicine**	**Unspecified specialties**	***p*-value between all the specialties and competencies**^**a**^	***p*-value Public health with BS vs other specialties**^**a**^
	**N (%)**	**N (%)**	**N (%)**	**N (%)**	**N (%)**	**N (%)**	**N (%)**		
**Category 1: Content and Context**									
**Science and Practice domain**									
Low competency	20 (90.9%)	30 (68.2%)	8 (57.1%)	5 (71.4%)	7 (100%)	1 (50.0%)	9 (64.3%)	0.056	**0.042**
High competency	2 (9.1%)	14 (31.8%)	6 (42.9%)	2 (28.6%)	0 (0.0%)	1 (50.0%)	5 (35.7%)		
**Promoting Health domain**									
Low competency	21 (95.5%)	28 (63.6%)	9 (64.3%)	3 (42.9%)	7 (100%)	0 (0.0%)	9 (64.3%)	**0.001**	**0.005**
High competency	1 (4.5%)	16 (36.4%)	5 (35.7%)	4 (57.1%)	0 (0.0%)	2 (100%)	5 (35.7%)		
**Law, Policies, and Health Security domain**									
Low competency	19 (86.4%)	28 (63.6%)	9 (64.3%)	6 (85.7%)	6 (85.7%)	0 (0.0%)	7 (50.0%)	**0.036**	0.055
High competency	3 (13.6%)	16 (36.4%)	5 (35.7%)	1 (14.3%)	1 (14.3%)	2 (100%)	7 (50.0%)		
**One Health and Health Security domain**									
Low competency	19 (86.4%)	31 (70.5%)	10 (71.4%)	4 (57.1%)	6 (85.7%)	0 (0.0%)	11 (78.6%)	0.121	0.155
High competency	3 (13.6%)	13 (29.5%)	4 (28.6%)	3 (42.9%)	1 (14.3%)	2 (100%)	3 (21.4%)		
**Category 2: Relations and Interactions**									
**Leadership and Systems Thinking domain**									
Low competency	20 (90.9%)	29 (65.9%)	11 (78.6%)	3 (42.9%)	7 (100%)	1 (50.0%)	7 (50.0%)	**0.008**	**0.029**
High competency	2 (9.1%)	15 (34.1%)	3 (21.4%)	4 (57.1%)	0 (0.0%)	1 (50.0%)	7 (50.0%)		
**Collaboration and Partnerships domain**									
Low competency	21 (95.5%)	25 (56.8%)	11 (78.6%)	2 (28.6%)	6 (85.7%)	0 (0%)	6 (42.9%)	** < 0.001**	**0.001**
High competency	1 (4.5%)	19 (43.2%)	3 (21.4%)	5 (71.4%)	1 (14.3%)	2 (100%)	8 (57.1%)		
**Communication, Culture, and Advocacy domain**									
Low competency	19 (86.4%)	28 (63.6%)	10 (71.4%)	3 (42.9%)	7 (100%)	2 (100%)	6 (42.9%)	**0.012**	0.055
High competency	3 (13.6%)	16 (36.4%)	4 (28.6%)	4 (57.1%)	0 (0%)	0 (0%)	8 (57.1%)		
**Category 3: Performance and achievements**									
**Governance and Resource Management domain**									
Low competency	21 (95.5%)	29 (65.9%)	10 (71.4%)	4 (57.1%)	7 (100%)	1 (50%)	7 (50%)	**0.005**	**0.008**
High competency	1 (4.5%)	15 (34.1%)	5 (28.6%)	3 (42.9%)	0 (0.0%)	1 (50%)	7 (50%)		
**Organizational Literacy and Adaptability domain**									
Low competency	19 (86.4%)	25 (56.8%)	9 (64.3%)	4 (57.1%)	5 (71.4%)	1 (50%)	6 (42.9%)	0.103	**0.016**
High competency	3 (13.6%)	19 (43.2%)	5 (35.7%)	3 (42.9%)	2 (28.6%)	1 (50%)	8 (57.1%)		
**Professional Development and Reflective Ethical Practice domain**									
Low competency	20 (90.9%)	28 (63.6%)	9 (64.3%)	3 (42.9%)	6 (85.7%)	1 (50%)	9 (64.3%)	0.067	**0.019**
High competency	2 (9.1%)	16 (36.4%)	5 (35.7%)	4 (57.1%)	1 (14.3%)	1 (50%)	5 (35.7%)		

^a^Numbers in bold indicate statistically significant results

In Category 1 (Content and Context), the results showed statistically significant differences between public health versus other specialties in the domains of “Science and Practice” (*p* = 0.042) and “Promoting Health” (*p* = 0.005), with the holders of a BS in public health degree declaring being less competent than their counterparts from other specialties. A significant association was found between all specialties and the domains of “Promoting Health” (*p* = 0.001), where the nursing specialty scored higher than other specialties. In addition, medical doctors showed a higher competency in Law, Policies, and Health Security domain than other health professionals (*p* = 0.036).

In Category 2 (Relations and Interactions), statistically significant differences in knowledge were found in all domains between all specialties and between public health specialists versus all others (*p* < 0.05), except for a borderline difference (*p* = 0.055) when comparing the level of competency in “Communication, Culture, and Advocacy” between public health and other specialties. Public health degree holders declared being less competent than other public health professionals, with nurses being more competent than all others in this domain.

In Category 3 (performance and achievements), the results showed statistically significant differences between public health versus other specialties (*p* < 0.05), where public health degree holders were also less competent than professionals from other specialties. Medical doctors seemed more competent than other practitioners in the domain of “Governance and Resource Management” (*p* = 0.005).

However, the results showed non-significant differences in the declared level of competencies in Category 1 (Content and Context), in the domain of “One-Health and Health Security” between all specialties (*p* = 0.121) and between public health versus all others (*p* = 0.155).

### Feedback on the main competencies needed for public health practice

Table 5 highlights the feedback agreement of the participants on the main competencies needed for public health practitioners based on their experience. The vast majority of participants (90.9%) agreed that “having foundational training in a health discipline” is a priority. Less than half of them (43.9%) considered that “performing intuitively and only occasionally need deliberation” is a priority for public health practitioners.

**Table 5:** Feedback of participants agreement on the main competencies that are needed for public health practitioners

	**Frequency (%)**
Focus on the central aspects of a problem	51 (77.3%)
Perform intuitively and only occasionally need deliberation	29 (43.9%)
Reflect on how the system works	57 (86.4%)
Assess the quality of the work done in their organization	59 (89.4%)
Assume leadership roles	53 (80.3%)
Develop strategies and assign leadership responsibilities to others	55 (83.3%)
Have substantial authority and responsibility	56 (84.8%)
Supervise multiple tiers of staff	50 (75.8%)
Make decisions via intuition and analytical thinking	55 (83.3%)
See the situation and the interconnectedness of the decisions they make	58 (87.9%)
Have supervisory responsibility	51 (77.3%)
Have foundational training in a health discipline	60 (90.9%)
Rely heavily on their core public health competencies	53 (80.3%)
Recognize that complex work requires non-routine decision-making, to which hard and fast rules do not clearly apply	51 (77.3%)
Supervise smaller groups of staff	43 (65.2%)

### Multivariate analysis

**Fig. 1:**
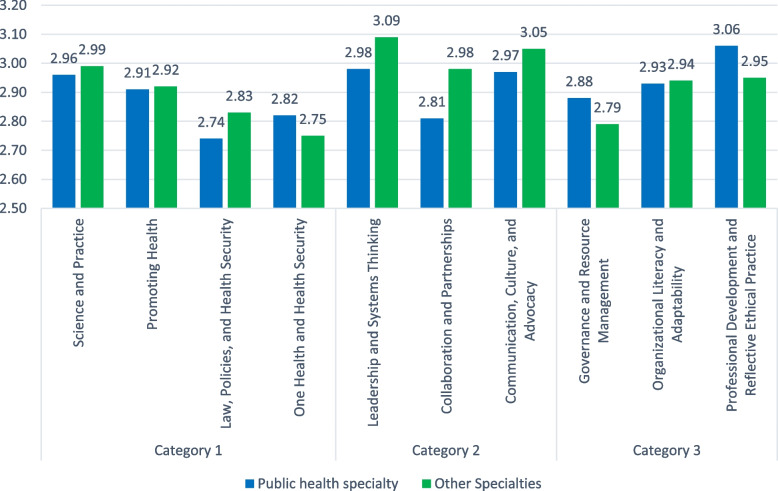
Adjusted means of health competency domains according to the type of specialty (public health vs. other specialties). No significant difference between public health and other specialties in self-declared competency domains with *p* > 0.05

Table 6 shows no significant associations between baseline specialties and self-declared competencies, while the latter were sometimes affected by sociodemographic characteristics (Fig. 1).

**Table 6:** Association between the public health competencies score by category and public health specialty vs other specialties

	**Beta**	***p*-value**	**95% Confidence Interval**	
			**Lower Bound**	**Upper Bound**
**Category 1: Content and Context**				
**Science and Practice**				
Gender (females vs males)	0.467	0.080	-0.058	0.992
Years of experience (1–5 years)	-0.082	0.721	-0.543	0.379
Years of experience (6–10 years)	0.613	0.050	0.0001	1.225
Area of practice academia	-0.211	0.286	-0.603	0.181
Area of practice medical setting	-0.104	0.532	-0.434	0.227
**Area of practice research epidemiology**	**0.412**	**0.039**	**0.022**	**0.801**
Area of practice NGO	0.007	0.966	-0.327	0.341
Area of practice MOPH	0.049	0.784	-0.305	0.403
Area of practice fresh graduate	0.181	0.371	-0.222	0.583
**Area of residence Mont Lebanon**	**-0.458**	**0.026**	**-0.858**	**-0.058**
Area of residence North	-0.607	0.143	-1.426	0.212
Area of residence South	0.195	0.580	-0.508	0.899
Area of residence Bekaa	0.195	0.689	-0.780	1.170
Specialty (public health vs others^a^)	-0.047	0.795	-0.409	0.315
**Promoting Health**				
**Gender (females vs males)**	**0.637**	**0.042**	**0.024**	**1.251**
Years of experience (1–5 years)	-0.186	0.491	-0.724	0.352
Years of experience (6–10 years)	0.474	0.189	-0.241	1.189
Area of practice academia	0.038	0.868	-0.420	0.496
Area of practice medical setting	0.083	0.668	-0.303	0.469
Area of practice research epidemiology	-0.273	0.235	-0.728	0.183
Area of practice NGO	0.173	0.379	-0.218	0.563
Area of practice MOPH	0.099	0.633	-0.314	0.512
Area of practice fresh graduate	-0.019	0.936	-0.489	0.451
Area of residence Mont Lebanon	-0.467	0.050	-0.935	-0.005
Area of residence North	-0.171	0.721	-1.127	0.785
Area of residence South	0.093	0.822	-0.729	0.914
Area of residence Bekaa	0.006	0.992	-1.133	1.145
Specialty (public health vs others^a^)	-0.040	0.851	-0.463	0.384
**Law, Policies, and Health Security**				
Gender (females vs males)	0.360	0.215	-0.216	0.935
**Years of experience (1–5 years)**	**-0.625**	**0.016**	**-1.130**	**-0.120**
Years of experience (6–10 years)	-0.223	0.508	-0.894	0.448
Area of practice academia	0.147	0.496	-0.283	0.576
Area of practice medical setting	-0.118	0.515	-0.480	0.244
Area of practice research epidemiology	-0.005	0.983	-0.432	0.423
Area of practice NGO	0.104	0.571	-0.262	0.470
**Area of practice MOPH**	**0.457**	**0.022**	**0.069**	**0.845**
Area of practice fresh graduate	0.040	0.857	-0.401	0.481
**Area of residence Mont Lebanon**	**-0.670**	**0.003**	**-1.108**	**-0.232**
Area of residence North	-0.259	0.564	-1.156	0.637
Area of residence South	0.156	0.687	-0.615	0.927
Area of residence Bekaa	0.005	0.993	-1.064	1.073
Specialty (public health vs others^a^)	-0.012	0.951	-0.409	0.385
**One Health and Health Security**				
Gender (females vs males)	0.499	0.092	-0.084	1.083
Years of experience (1–5 years)	-0.215	0.403	-0.727	0.297
Years of experience (6–10 years)	0.503	0.144	-0.177	1.184
Area of practice academia	-0.109	0.619	-0.544	0.327
Area of practice medical setting	0.191	0.302	-0.176	0.558
Area of practice research epidemiology	-0.150	0.492	-0.583	0.284
Area of practice NGO	0.200	0.285	-0.171	0.571
**Area of practice MOPH**	**0.511**	**0.012**	**0.117**	**0.904**
Area of practice fresh graduate	-0.130	0.562	-0.577	0.317
**Area of residence Mont Lebanon**	**-0.646**	**0.005**	**-1.091**	**-0.202**
Area of residence North	-0.395	0.388	-1.304	0.515
Area of residence South	-0.305	0.437	-1.087	0.477
Area of residence Bekaa	0.203	0.708	-0.880	1.286
Specialty (public health vs others^a^)	0.077	0.702	-0.326	0.480
**Category 2: Relations and Interactions**				
**Leadership and Systems Thinking**				
Gender (females vs males)	0.527	0.090	-0.084	1.138
Years of experience (1–5 years)	-0.171	0.525	-0.708	0.366
Years of experience (6–10 years)	0.557	0.123	-0.156	1.270
Area of practice academia	-0.428	0.065	-0.884	0.028
Area of practice medical setting	-0.327	0.094	-0.712	0.057
Area of practice research epidemiology	0.105	0.643	-0.349	0.559
Area of practice NGO	0.375	0.059	-0.015	0.764
Area of practice MOPH	0.163	0.432	-0.250	0.575
Area of practice fresh graduate	0.048	0.838	-0.421	0.517
**Area of residence Mont Lebanon**	**-0.711**	**0.003**	**-1.177**	**-0.245**
**Area of residence North**	**-1.405**	**0.005**	**-2.358**	**-0.452**
Area of residence South	-0.393	0.340	-1.212	0.426
Area of residence Bekaa	0.241	0.671	-0.894	1.376
Specialty (public health vs others^a^)	-0.051	0.808	-0.473	0.371
**Collaboration and Partnerships**				
**Gender (females vs males**^**a**^	**0.649**	**0.032**	**0.060**	**1.239**
Years of experience (1–5 years)	-0.296	0.257	-0.813	0.222
Years of experience (6–10 years)	0.104	0.763	-0.584	0.792
Area of practice academia	0.041	0.851	-0.399	0.482
Area of practice medical setting	-0.113	0.545	-0.484	0.259
Area of practice research epidemiology	-0.183	0.406	-0.621	0.255
Area of practice NGO	0.325	0.089	-0.051	0.700
Area of practice MOPH	0.319	0.113	-0.078	0.717
Area of practice fresh graduate	-0.181	0.424	-0.633	0.271
**Area of residence Mont Lebanon**	**-0.491**	**0.033**	**-0.941**	**-0.042**
**Area of residence North**	**-1.037**	**0.028**	**-1.956**	**-0.117**
Area of residence South	0.035	0.930	-0.756	0.825
Area of residence Bekaa	-0.256	0.640	-1.351	0.838
Specialty (public health vs others^a^)	-0.199	0.332	-0.606	0.208
**Communication, Culture, and Advocacy**				
**Gender (females vs males)**	**0.773**	**0.011**	**0.184**	**1.361**
Years of experience (1–5 years)	-0.421	0.108	-0.938	0.095
Years of experience (6–10 years)	0.076	0.824	-0.610	0.763
Area of practice academia	-0.121	0.581	-0.561	0.318
Area of practice medical setting	-0.110	0.554	-0.480	0.260
Area of practice research epidemiology	-0.031	0.886	-0.469	0.406
Area of practice NGO	0.307	0.106	-0.067	0.682
Area of practice MOPH	0.104	0.600	-0.292	0.501
Area of practice fresh graduate	-0.104	0.644	-0.555	0.347
Area of residence Mont Lebanon	-0.366	0.107	-0.815	0.082
**Area of residence North**	**-1.314**	**0.006**	**-2.232**	**-0.396**
Area of residence South	-0.430	0.279	-1.219	0.359
Area of residence Bekaa	0.305	0.577	-0.788	1.398
Specialty (public health vs others^a^)	-0.155	0.448	-0.561	0.252
**Category 3: Performance and achievements**				
**Governance and Resource Management**				
Gender (females vs males)	0.458	0.142	-0.159	1.075
Years of experience (1–5 years)	-0.442	0.108	-0.983	0.100
Years of experience (6–10 years)	0.004	0.992	-0.716	0.724
Area of practice academia	-0.200	0.387	-0.661	0.260
Area of practice medical setting	-0.112	0.566	-0.500	0.277
Area of practice research epidemiology	-0.070	0.760	-0.529	0.388
Area of practice NGO	0.062	0.754	-0.331	0.455
Area of practice MOPH	0.368	0.082	-0.048	0.784
Area of practice fresh graduate	-0.248	0.297	-0.721	0.225
**Area of residence Mont Lebanon**	**-0.522**	**0.030**	**-0.992**	**-0.052**
Area of residence North	-0.845	0.084	-1.808	0.117
Area of residence South	0.013	0.976	-0.815	0.840
Area of residence Bekaa	0.540	0.349	-0.606	1.686
Specialty (public health vs others^a^)	0.046	0.830	-0.380	0.472
**Organizational Literacy and Adaptability**				
Gender (females vs males)	0.564	0.090	-0.091	1.218
Years of experience (1–5 years)	-0.118	0.682	-0.692	0.456
Years of experience (6–10 years)	0.527	0.172	-0.236	1.291
Area of practice academia	-0.042	0.863	-0.531	0.446
Area of practice medical setting	-0.180	0.384	-0.592	0.232
Area of practice research epidemiology	-0.026	0.914	-0.512	0.460
Area of practice NGO	-0.041	0.844	-0.458	0.376
Area of practice MOPH	0.141	0.523	-0.300	0.583
Area of practice fresh graduate	-0.267	0.291	-0.768	0.235
**Area of residence Mont Lebanon**	**-0.548**	**0.032**	**-1.047**	**-0.050**
**Area of residence North**	**-1.249**	**0.017**	**-2.270**	**-0.229**
Area of residence South	0.431	0.329	-0.446	1.308
Area of residence Bekaa	-0.161	0.791	-1.377	1.054
Specialty (public health vs others^a^)	-0.012	0.958	-0.464	0.440
**Professional Development and Reflective Ethical Practice**				
**Gender (females vs males)**	**0.763**	**0.024**	**0.105**	**1.420**
Years of experience (1–5 years)	-0.235	0.417	-0.812	0.342
**Years of experience (6–10 years)**	**0.834**	**0.034**	**0.067**	**1.601**
Area of practice academia	-0.210	0.395	-0.700	0.281
Area of practice medical setting	-0.232	0.265	-0.646	0.182
Area of practice research epidemiology	-0.131	0.592	-0.620	0.357
Area of practice NGO	0.209	0.322	-0.210	0.627
Area of practice MOPH	-0.070	0.753	-0.513	0.373
Area of practice fresh graduate	-0.312	0.220	-0.815	0.192
**Area of residence Mont Lebanon**	**-0.686**	**0.008**	**-1.187**	**-0.185**
**Area of residence North**	**-1.312**	**0.013**	**-2.337**	**-0.287**
Area of residence South	-0.371	0.402	-1.252	0.510
Area of residence Bekaa	0.057	0.926	-1.164	1.278
Specialty (public health vs others^a^)	0.140	0.539	-0.314	0.593

In the global model, the independent variable is “specialty” (public health vs others*). Covariates are gender, years of experience, area of residence and area of practice

^a^Reference group

There were no statistically significant differences between public health practitioners and all others for any of these competencies (*p* > 0.05 for all).

### Category 1 (Content and Context)

Practicing as a research epidemiologist (Beta = 0.412, *p* = 0.039) was significantly associated with a higher “Science and Practice” score. Female gender (beta = 0.637, *p* = 0.042) was significantly associated with a higher “Promoting Health” score. Working in the Ministry of Public Health was significantly associated with higher “Law, Policies, and Health Security” (Beta = 0.457, *p* = 0.022) and higher “One-Health and Health Security” scores (Beta = 0.511, *p* = 0.012). Having an experience of 1–5 years (Beta = -0.625, *p* = 0.016) was significantly associated with lower “Law, Policies, and Health Security” scores. Living in Mount Lebanon was significantly associated with lower scores in all Category 1 competencies.

### Category 2 (Relations and Interactions)

Participants living in the Mount Lebanon and North regions scored significantly lower in three competencies (Leadership and Systems Thinking, Collaboration and Partnerships, and Communication, Culture, and Advocacy). Female gender was significantly associated with higher “Collaboration and Partnerships” and “Communication, Culture, and Advocacy” scores.

### Category 3 (Performance and Achievements)

Living in Mount Lebanon was significantly associated with lower scores in three competencies (Governance and Resource Management, Organizational Literacy and Adaptability, and Professional Development and Reflective Ethical Practice). Also, participants from North Lebanon scored significantly lower on “Organizational Literacy and Adaptability” and “Professional Development and Reflective Ethical Practice”. Being a female (Beta = 0.763, *p* = 0.024) and having an experience of 6–10 years (Beta = 0.834, *p* = 0.034) were significantly associated with higher “Professional Development and Reflective Ethical Practice” scores.

## Discussion

Our study is the first to validate a tool to assess self-declared public health competencies, namely the WHO-ASPHER framework. The framework comprises three categories, i.e., 1) Content and Context, 2) Relations and Interactions, and 3) Performance and Achievements, each divided into domains that include many items. The factor analysis for scale domains showed that Barlett’s test of sphericity was significant (*p* < 0.001), high loadings of items on factors, and Cronbach’s alpha values of more than 0.9 in all three categories, indicating appropriate validity and reliability. These results show the possibility of applying a European framework in a developing country, which can be considered an innovation in the Lebanese context in the absence of a national framework. Our results are also close to those of Zwanikken and collaborators, who used Delphi rounds with experts and alumni feedback to validate their framework in low- and middle-income countries [45]; they came up with domains of a different structure than ours, but the content is overall comparable. The WHO-ASPHER framework can thus be used in Lebanon and would also allow benchmarking at the international level.

In Lebanon, the suggested framework would thus allow public health professionals to self-evaluate their proficiency level in different domains and determine the gaps in knowledge that need strengthening. Investment in the public health workforce is more highly mandated now than ever [26, 46, 47]. The COVID-19 pandemic highlighted global weaknesses in the health systems against the threat of communicable diseases and disease outbreaks [26, 48]. Consequently, strengthening public health capacity and services has become a global priority [9, 26, 49–51], and the core competencies in the public health framework allow professionals to reach this goal [26] and help identify the essential individual attributes required to fulfill their role [52, 53]. Indeed, the Institute of Medicine (IOM) and other academic, governmental and non-governmental institutions emphasized the need to enhance academic preparedness to meet the 21^st^-century public health challenges [51, 54–62].

The suggested framework would also help stakeholders, such as policy-makers, educational institutions, and public health institutes [26], develop context-specific competency measures to improve education, performance, capacity-building, analysis, and monitoring, in addition to planning and investment [26]. Our study validated the framework to offer an evidence-based, comprehensive template that helps the public health practitioner identify the domains that need strengthening and guides the academic sector to plan a curriculum that meets current and future public health challenges.

Data analysis of the survey showed that the perceived level of competencies was significantly different between the public health professionals and other health professionals with activities in public health. Graduates with public health degrees declared a lower competency level than other health professionals; the latter had variable competency levels in different domains, depending on the health specialty. It is noteworthy that multivariate analysis showed that differences were no longer significant, likely due to the low sample size.

Our findings also revealed that public health core competencies and workforce requirements are not yet well delineated at the national level. All respondents from different educational backgrounds scored low in most public health categories, mainly science and practice. Other studies reported similar results, highlighting the need to call for action to build a public health workforce [56, 63, 64]. Most participants agreed that foundational training in a health discipline is the main competency needed for public health professionals. These findings shed light on the existing capacity and future training requirements to strengthen education tailored to national needs [26].

Studies similar to ours using a formulated framework or survey showed that the main gaps were communication, budgeting and financial planning [29–31], systems thinking [30, 31, 65], policy development [29, 65, 66], and other management skills [29, 31, 65] among surveyed participants. Other gaps included developing a vision for a healthier community [30]. The level of competencies was significantly different between public health professionals and other health professionals with activities in public health. Creating a public health workforce that delivers essential services in all domains of the three core competency categories is critical and challenging at the same time. According to the WHO-ASPHER, professionals are expected to demonstrate a subset of their competencies related to their role [26].

This study offers baseline data to conduct in-depth research across Lebanon, including public health professionals from multiple disciplines and universities with variable levels of expertise and practice in the field. Based on these findings, building a highly-performing Lebanese public health workforce, linking education to practice, and enhancing cross-disciplinary collaboration would help design an academic curriculum for excellence in public health practice. This study also highlighted the importance of setting national guidelines for public health workforce planning and policy-supporting workforce development while addressing the gaps and pitfalls in the field. The guidelines should be tailored to the local requirements to set targeted objectives and plan a joint action based on the adapted WHO-ASPHER framework to the national context. Other countries can benefit from this framework to allow benchmarking, follow-up, and collaborative international action plans for health policy-making to improve competencies in public health.

This study would be the ground for identifying workforce misdistribution, inefficiencies, performance evaluation, and quality assurance to build a workforce for excellence. To reach this point, strategies related to public health education and the workforce are necessary, based on further assessment of the Lebanese context; authorities, academia, professionals, and other stakeholders should join efforts to develop and implement such strategies.

### Strengths and limitations

Our study is the first to validate the scale for self-assessment of public health core competencies. It offers a valuable tool for academia and public health professionals to self-assess the level of public health proficiency and orientate continuous education needs for professional development on an individual level while also offering evidenced data for curriculum review and identification of training needs in the academic sector.

The main limitation of this study is the low number of participants per specialty; thus, larger-scale studies are warranted to confirm these descriptive results. The survey was web-based, which may be amenable to sampling and response bias, given in particular that the population of public health professionals is large and unclearly defined. Moreover, when diffusing the questionnaire on social media, most accounts were open; thus, the exact number of potential participants who received the survey link could not be assessed. Respondents were mainly females with one to five years of experience, which hampers the generalizability of the results. Participants self-rated their level of competency in public health services, reflecting their perception only and leading to reporting bias. However, the study design and method used are common to other tool validation studies.

## Conclusion

Our study offered a validated tool for academia and public health professionals based on the WHO-ASPHER framework to self-assess the level of public health proficiency and guide continuous education needs for professional development. Data findings also showed variability of self-declared gaps in knowledge and skills, suggesting a need to review the national public health education programs. This study calls for close collaboration between academia and health policy-makers to strengthen public health by addressing national gaps and needs while joining forces with international health organizations to improve the global readiness for future health hurdles.

## Supplementary Information

**Additional file 1.**

## Data Availability

The datasets generated and/or analysed during the current study are available in the INSPECT-LB repository, https://inspect-lb.org/assessing-self-reported-core-competencies-of-public-health-practitioners-in-lebanon-using-the-who-aspher-validated-scale-a-pilot-study/.
