# Supplementary material for: Assessing self-reported core competencies of public health practitioners in Lebanon using the WHO-ASPHER validated scale: a pilot study
Source: BMC Med Educ. 2022 Dec 20;22:882. doi: 10.1186/s12909-022-03940-4 (PMC9763804; doi:10.1186/s12909-022-03940-4)
Supplement: Supplementary file 1 — Additional file 1. [file 12909_2022_3940_MOESM1_ESM.pdf]

# Assessment of Core Competencies, Knowledge, and Skills of Public Health Practitioners Upon Graduation

Dear participants,

You are invited to participate in a research about core competencies, knowledge, and skills of public health practitioners acquired upon graduation.

This research conducted by a group of academic researchers aims to determine the domains that need strengthening for a high-performing public health system.

The information gathered in this 15-minute survey is anonymous and will be treated confidentially.

We thank you in advance for your time,

The research team

**Informed consent**

Please check all the boxes to proceed to the survey

☐ I have read and understood the above information

☐ I understand that my participation is voluntary

☐ I understand that my data will be kept confidential

☐ I agree to participate in this study

## DEMOGRAPHIC CHARACTERISTICS

1. **Age:** ______________________

2. **Gender:** ☐Male ☐Female

3. **Area of residence**
   ☐Beirut ☐Mount Lebanon ☐North ☐South ☐Beqaa

4. **Are you specialized in any of the following fields? (Check all that apply)**
   ☐Public health
   ☐Medicine
   ☐Pharmacy
   ☐Nursing
   ☐Nutrition
   ☐Other. Please specify: ______________________

5. **Your area of public health practice is: (Check all that apply)**
   ☐Academia
   ☐Medical setting
   ☐Research and Epidemiology
   ☐Non-governmental organization
   ☐Ministry of public health
   ☐I graduated recently, I do not currently work

6. **Years of experience in the field**
   ☐1-5 years ☐6-10 years ☐More than 10 years

## PUBLIC HEALTH ESSENTIAL OPERATIONS

7. **Rate your knowledge and skills to perform the following essential public health operations upon graduation, using the following scale:**
   None: I am unaware or have very little knowledge of the skill
   Aware: I have heard of, but have limited knowledge or ability to apply the skill
   Knowledgeable: I am comfortable with my knowledge or ability to apply the skill
   Proficient: I am very comfortable, I am an expert, or could teach this skill to others

| | None | Aware | Knowledgeable | Proficient |
|---|---|---|---|---|
| Surveillance of population health and well-being | | | | |
| Monitoring and response to health hazards and emergencies | | | | |

| | | | | |
|---|---|---|---|---|
| Health protection, including environmental, occupational, food safety, and other | | | | |
| Health promotion, including action to address social determinants and health inequity | | | | |
| Disease prevention, including early detection of illness | | | | |
| Assuring governance for health and well-being | | | | |
| Assuring a sufficient and competent health workforce | | | | |
| Assuring sustainable organizational structures and financing | | | | |
| Advocacy communication and social mobilization for health | | | | |
| Advancing public health research to inform policy and practice | | | | |

## ASSESSMENT OF THE LEVEL OF PUBLIC HEALTH WORKFORCE COMPETENCY

**Category 1: Content and Context**

This category encompasses four sections: 1) Science and Practice; 2) Promoting Health; 3) Law, Policies, and Health Security; 4) One Health and Health Security

8. **In the field of Science and Practice (epidemiology, demography, biostatistics, health indicators, population health, and inequalities), rate your ability to perform the following upon graduation, using a scale from none to proficient:**

None: I am unaware or have very little knowledge of the skill

Aware: I have heard of, but have limited knowledge or ability to apply the skill

Knowledgeable: I am comfortable with my knowledge or ability to apply the skill

Proficient: I am very comfortable, I am an expert, or could teach this skill to others

| | None | Aware | Knowledgeable | Proficient |
|---|---|---|---|---|
| Describe the features of national demographic structure and its implications for public health | | | | |
| Determine the key features of the epidemiology, trends, incidence, and prevalence of the significant diseases in Lebanon | | | | |
| Use vital statistics and health indicators | | | | |
| Identify the strengths and weaknesses of routine data and use these data as part of the complex assessment of population needs | | | | |

| | | | | |
|---|---|---|---|---|
| Retrieve, analyze, and appraise evidence from all data sources to support decision-making | | | | |
| Address the main health needs of the Lebanese population | | | | |
| Establish and monitor indicators of population health | | | | |
| Compare and assess the needs and services provided to meet health needs | | | | |
| Contribute to or lead community-based health needs assessments | | | | |
| Review routine data and the literature to what actions should be taken to meet health needs | | | | |
| Design and conduct qualitative and/or quantitative research that adds to the evidence base for public health practice | | | | |
| Show a high level of knowledge of research methods and analysis techniques | | | | |
| Evaluate local public health services and interventions, applying sound methods based on recognized evaluation models | | | | |

9. **In the field of health promotion (education, promotion through social participation, citizen empowerment, health needs assessment, screening and secondary prevention, evaluation of health interventions programs), rate your ability to perform the following upon graduation, using a scale from none to proficient:**
None: I am unaware or have very little knowledge of the skill
Aware: I have heard of, but have limited knowledge or ability to apply the skill
Knowledgeable: I am comfortable with my knowledge or ability to apply the skill
Proficient: I am very comfortable, I am an expert, or could teach this skill to others

| | None | Aware | Knowledgeable | Proficient |
|---|---|---|---|---|
| Use health promotion theory and the options for delivering health-promotion initiatives | | | | |
| Raise health literacy | | | | |
| Promote the health of the public using evidence-based methods | | | | |
| Ensure that health education and health literacy activities are informed by evidence and/or theory | | | | |
| Contribute to the evaluation of the effectiveness of activities to promote health to lead changes at various levels across different sectors | | | | |

| | | | | |
|---|---|---|---|---|
| Use appropriate methods to foster citizens empowerment and community engagement | | | | |
| Consult with the public to engage meaningful decision-making that represents the wider societal views | | | | |
| Challenge incorrect information delivered to the public using a wide range of approaches, including communication with the media and politicians | | | | |
| Know the rationale for screening programs and the basis of secondary prevention in my country | | | | |
| Focus on disease prevention, reduction of inequalities, and equity in access to health services | | | | |
| Explore the underlying causes of morbidity and mortality, and recommendations to address these determinants of health and health services | | | | |

10. **In the field of law, policy, and ethics (policy development and planning, knowledge of national and international laws, ethical practice, and decision-making), rate your ability to perform the following upon graduation, using a scale from none to proficient:**
None: I am unaware or have very little knowledge of the skill
Aware: I have heard of, but have limited knowledge or ability to apply the skill
Knowledgeable: I am comfortable with my knowledge or ability to apply the skill
Proficient: I am very comfortable, I am an expert, or could teach this skill to others

| | None | Aware | Knowledgeable | Proficient |
|---|---|---|---|---|
| Comply with the legislation and professional codes of practice associated In my interaction with others | | | | |
| Understand and apply the laws and regulations directly or indirectly applicable to the practice of public health in Lebanon | | | | |
| Apply scientific principles and concepts to inform discussion of health-related fiscal, social, and political issues | | | | |
| Compare and contrast health and social service delivery systems between countries | | | | |
| Contribute to the delivery of equitable and effective health care and policies to improve the health of the public | | | | |
| Develop and implement strategies based on relevant evidence, legislation, emergency planning, procedures regulations, and policies | | | | |

| | | | | |
|---|---|---|---|---|
| Maximize opportunities to protect and promote health and well-being using applied laws and regulations | | | | |

**11. In the field of one health and health security (human health, health protection, food safety, animal health, cross-border health, international health, global risks and threats, environmental health, and climate change), rate your ability to perform the following upon graduation, using a scale from none to proficient:**

None: I am unaware or have very little knowledge of the skill

Aware: I have heard of, but have limited knowledge or ability to apply the skill

Knowledgeable: I am comfortable with my knowledge or ability to apply the skill

Proficient: I am very comfortable, I am an expert, or could teach this skill to others

| | **None** | **Aware** | **Knowledgeable** | **Proficient** |
|---|---|---|---|---|
| Understand the local implications of the One Health approach and its global interconnectivity | | | | |
| Prevent risks and mitigate the health crises that originate at the interface between human, animals, and environments and affect the health of the population | | | | |
| Analyze critically the changing nature, key factors, and resources that shape One Health | | | | |
| Understand the One Health | | | | |
| Apply the International Health regulations to coordinate and develop strategic partnerships and resources in key sectors and disciplines for health security purposes | | | | |
| Promote occupational health and health and safety regulations and legislations | | | | |
| Apply the practical principles of food safety essential to public health | | | | |
| Use multisectoral evidence-based guidelines for preventing and controlling health risks and diseases | | | | |
| Comply with the requirements of both formal and informal surveillance systems and conduct risk assessment | | | | |
| Identify and assure minimum safety standards in delivering services | | | | |
| Identify and describe environmental determinants of health and connections between environmental protection and public health policy | | | | |
| Understand the impact of climate on health and the responsibility of public health for protecting the natural environment | | | | |

**Category 2: Relations and Interactions**

This category encompasses three sections: 1) Leadership and Systems Thinking; 2) Collaboration and Partnerships; 3) Communication, Culture, and Advocacy.

**12. In the field of leadership and systems thinking (vision, mission, strategy, leading change and innovation, people development, emotional intelligence, organizational learning, and development), rate your ability to perform the following upon graduation, using a scale from none to proficient:**

None: I am unaware or have very little knowledge of the skill

Aware: I have heard of, but have limited knowledge or ability to apply the skill

Knowledgeable: I am comfortable with my knowledge or ability to apply the skill

Proficient: I am very comfortable, I am an expert, or could teach this skill to others

| | None | Aware | Knowledgeable | Proficient |
|---|---|---|---|---|
| Motivate others to work toward common vision, program, and/or organizational goals | | | | |
| Facilitate the development of other leaders | | | | |
| Identify and support the roles and responsibilities of all team members, including external stakeholders | | | | |
| Demonstrate emotional intelligence and understand the impact of one's belief, values, and behaviors on decision-making and others' reactions | | | | |
| Show practicality, flexibility, and adaptability in working with others to achieve public health goals | | | | |
| Lead and work as part of an interdisciplinary team | | | | |
| Catalyze behavioral, and/or cultural changes | | | | |
| Support initiatives for change at the organization, community, or individual level | | | | |
| Understand principles of systems thinking to the improve delivery of public health services | | | | |

**13. In the field of collaboration and interactions (effective collaboration, building alliances and partnerships, working and building interdisciplinary and intersectoral networks), rate your ability to perform the following upon graduation, using a scale from none to proficient:**

None: I am unaware or have very little knowledge of the skill

Aware: I have heard of, but have limited knowledge or ability to apply the skill

Knowledgeable: I am comfortable with my knowledge or ability to apply the skill

Proficient: I am very comfortable, I am an expert, or could teach this skill to others

| | None | Aware | Knowledgeable | Proficient |
|---|---|---|---|---|
| Work across sectors in organizational structures at the national and international levels | | | | |
| Establish effective partnerships and understand the priorities and motivations of a wide range of stakeholders | | | | |
| Identify, connect, and manage relationships with stakeholders in interdisciplinary and intersectoral projects to improve public health services and goals | | | | |
| Build, maintain, and effectively use strategic alliances, coalitions, professional networks, and partnerships to plan and generate evidence implement programs | | | | |
| Evaluate partnerships and address barriers to successful collaboration to improve public | | | | |
| Understand and apply effective techniques for working with boards and governance | | | | |

**14. In the field of communication, culture, and advocacy (effective communication with the media, scientific communication, historical and cultural context, advocacy, and diplomacy), rate your ability to perform the following <u>upon graduation</u>, using a scale from none to proficient:**

None: I am unaware or have very little knowledge of the skill

Aware: I have heard of, but have limited knowledge or ability to apply the skill

Knowledgeable: I am comfortable with my knowledge or ability to apply the skill

Proficient: I am very comfortable, I am an expert, or could teach this skill to others

| | None | Aware | Knowledgeable | Proficient |
|---|---|---|---|---|
| Communicate strategically by defining target audience, listening, and developing audience-appropriate messaging | | | | |
| Understand the importance of communication at different organizational levels to gain political commitment, policy support, and social acceptance for a health goal or program | | | | |
| Convey information and complex scientific evidence in an understandable way to people | | | | |
| Recognize that social media and social marketing are increasingly important tools | | | | |
| Understand and apply cultural awareness and sensitivity in | | | | |

| | | | | |
|---|---|---|---|---|
| communication with diverse populations | | | | |
| Communicate with respect when representing professional opinions, and encourage other team members | | | | |
| Prepare a meeting agenda | | | | |
| Deliver administrative tasks that require communication within or across organizations | | | | |
| Advocate for health-related public policies and services to promote and protect human health and well-being | | | | |

**Category 3: Performance and achievements**

This category encompasses three sections: 1) Governance and Resource Management; 2) Professional Development and Reflective Ethical Practice; 3) Organizational Literacy and Adaptability

15. **In the field of governance and resource management (human resources management, financial planning, quality assurance, technical expertise, basic health economics, economic evaluation and analysis), rate your ability to perform the following upon graduation, using a scale from none to proficient:**

    None: I am unaware or have very little knowledge of the skill

    Aware: I have heard of, but have limited knowledge or ability to apply the skill

    Knowledgeable: I am comfortable with my knowledge or ability to apply the skill

    Proficient: I am very comfortable, I am an expert, or could teach this skill to others

| | None | Aware | Knowledgeable | Proficient |
|---|---|---|---|---|
| Apply knowledge of organizational systems, theories, and behaviors to set priorities for resources and achieve clear strategic goals and objectives | | | | |
| Manage people effectively by providing clarity on task responsibility, provide training, and give regular feedback on performance | | | | |
| Plan the allocation of work tasks to achieve the goals set by the organization | | | | |
| Develop descriptions to assure staffing at various organization levels | | | | |
| Conduct hiring interviews and evaluate candidates | | | | |
| Demonstrate knowledge of basic business practices and develop a business plan | | | | |
| Design proactively and monitor quality standards and apply quality improvement methods and tools to ensure that quality standards are met | | | | |

| | | | | |
|---|---|---|---|---|
| Use key accounting principles and financial management tools | | | | |
| Use risk management principles and programs | | | | |
| Understand and apply the principles of economic thinking in public health | | | | |
| Perform health evaluation and assessment of a given procedure, intervention strategy, or policy | | | | |

**16. In the field of organizational literacy and adaptability (use of technology, entrepreneurship, fundraising, digital health, social media), rate your ability to perform the following upon graduation, using a scale from none to proficient:**

None: I am unaware or have very little knowledge of the skill

Aware: I have heard of, but have limited knowledge or ability to apply the skill

Knowledgeable: I am comfortable with my knowledge or ability to apply the skill

Proficient: I am very comfortable, I am an expert, or could teach this skill to others

| | None | Aware | Knowledgeable | Proficient |
|---|---|---|---|---|
| Cope with uncertainty and manage work-related stress | | | | |
| Demonstrate persistence, perseverance, resilience, and the ability to call on personal resources and energy at time of challenge | | | | |
| Show entrepreneurial orientation through proactiveness, innovativeness, and risk-taking, generating potential solutions to critical situations | | | | |
| Adapt to changing professional environments and circumstances | | | | |
| Apply for available funding sources and opportunities | | | | |
| Respond to call for project applications and grants | | | | |
| Draft tender and project briefs | | | | |

**17. In the field of professional development and reflective ethical practice (ethical professional conduct, lifelong learning, professional and reflective practice), rate your ability to perform the following upon graduation, using a scale from none to proficient:**

None: I am unaware or have very little knowledge of the skill

Aware: I have heard of, but have limited knowledge or ability to apply the skill

Knowledgeable: I am comfortable with my knowledge or ability to apply the skill

Proficient: I am very comfortable, I am an expert, or could teach this skill to others

| | None | Aware | Knowledgeable | Proficient |
|---|---|---|---|---|
| Demonstrate willingness to pursue learning in public health | | | | |
| Address your own development needs based on career goals and required competencies | | | | |

| | | | | |
|---|---|---|---|---|
| Act according to ethical standards and norms with integrity, and promote professional accountability, social responsibility, and the public health good | | | | |
| Critically review and evaluate your own practices in relation with public health principles | | | | |
| Ensure the availability of development opportunities | | | | |
| Demonstrate an ability to understand and manage conflict-of-interest situations | | | | |
| Act and promote evidence-based professional practice | | | | |

## PUBLIC HEALTH PRACTITIONERS' FEEDBACK

**18. In your opinion, a qualified public health practitioner must be able to:**

| | Totally disagree | Disagree | Neutral | Agree | Totally agree |
|---|---|---|---|---|---|
| Focus on the central aspects of a problem | | | | | |
| Perform intuitively and only occasionally need deliberation | | | | | |
| Reflect on how the system works | | | | | |
| Assess the quality of the work done in their organization | | | | | |
| Assume leadership roles | | | | | |
| Develop strategies and assign leadership responsibilities to others | | | | | |
| Have substantial authority and responsibility | | | | | |
| Supervise multiple tiers of staff | | | | | |
| Make decisions via intuition and analytical thinking | | | | | |
| See the situation and the interconnectedness of the decisions they make | | | | | |
| Have supervisory responsibility | | | | | |
| Have foundational training in a health discipline | | | | | |
| Rely heavily on their core public health competencies | | | | | |
| Supervise smaller groups of staff | | | | | |

**19. What is the highest public health degree you earned?**

☐BS

☐MPH

☐MS

☐PhD

☐DPH

☐Other: ________

**20. Which university did you graduate from?**

☐Lebanese University

☐American University of Beirut

☐Saint Joseph University of Beirut

☐Beirut Arab University

☐University of Balamand

☐Notre Dame University

☐Université Sainte Famille

☐American University of Science and Technology

☐Modern University for Business and Science

☐Lebanese German University

☐I prefer not to say

☐Other: ________
